# Praziquantel coverage in schools and communities targeted for the elimination of urogenital schistosomiasis in Zanzibar: a cross-sectional survey

**DOI:** 10.1186/s13071-015-1244-0

**Published:** 2016-01-04

**Authors:** Stefanie Knopp, Bobbie Person, Shaali M. Ame, Said M. Ali, Juma Muhsin, Saleh Juma, Iddi S. Khamis, Muriel Rabone, Lynsey Blair, Alan Fenwick, Khalfan A. Mohammed, David Rollinson

**Affiliations:** Wolfson Wellcome Biomedical Laboratories, Department of Life Sciences, Natural History Museum, Cromwell Road, London, SW7 5BD UK; Department of Epidemiology and Public Health, Swiss Tropical and Public Health Institute, P.O. Box, CH–4002, Basel, Switzerland; University of Basel, P.O. Box, CH–4003, Basel, Switzerland; Schistosomiasis Consortium for Operational Research and Evaluation, University of Georgia, Athens, GA 30602 USA; Public Health Laboratory Ivo de Carneri, Ministry of Health, P.O. Box 122, Chake-Chake, Pemba United Republic of Tanzania; Helminth Control Laboratory Unguja, Ministry of Health, P.O. Box 236, Zanzibar, United Republic of Tanzania; Schistosomiasis Control Initiative, Department of Infectious Disease Epidemiology, Faculty of Medicine, VB1 Norfolk Place, St. Mary’s Campus, London, UK

**Keywords:** Compliance, Coverage, Elimination, Mass drug administration, Preventive chemotherapy, *Schistosoma haematobium*, Zanzibar

## Abstract

**Background:**

Biannual mass drug administration (MDA) with praziquantel and additional interventions to eliminate urogenital schistosomiasis has been implemented on the Zanzibar islands, United Republic of Tanzania, since 2012. We aimed to assess the coverage of school-based treatment (SBT) and community-wide treatment (CWT), to validate the coverage reported by the Zanzibar Ministry of Health (MoH) and to identify reasons for non-compliance.

**Methods:**

We conducted a post-MDA cross-sectional survey in 93 schools and 92 communities on Pemba and Unguja islands in early 2014, 3–5 months after the last MDA round. Pupils and adults were asked whether they had received and taken the praziquantel treatment provided in the last SBT or CWT, respectively, and the observed and reported coverage were compared. Reasons for non-compliance were recorded in a pretested questionnaire and assessed in qualitative interviews. Urine samples of participants were examined for S*chistosoma haematobium* eggs with a single urine filtration.

**Results:**

Around 8000 pupils and 4000 adults were included in the analysis. Our survey revealed a SBT coverage of 85.2 % in Pemba and of 86.9 % in Unguja, which was in line with MoH reports from Pemba (84.3 %) and higher than reports from Unguja (63.9 %). However, 15 among the 48 schools surveyed in Unguja had not received SBT. Among the interviewed adults, 53.6 % in Pemba and 64.9 % in Unguja had received praziquantel during CWT, which was less than the 59.0 % and 67.7 %, respectively, indicated by MoH reports. Moreover, only 43.8 % and 54.0 % of adults in Pemba and Unguja, respectively, had taken all the tablets as recommended. The main reasons for not receiving or taking praziquantel were absence during CWT, no drug distributor coming, being busy, fear of adverse events, pregnancy, breastfeeding or feeling healthy.

**Conclusion:**

To increase coverage and compliance in Zanzibar, SBT should target all schools and mobilization, sensitization and implementation of the CWT need to be improved. To reach elimination of urogenital schistosomiasis transmission in Zanzibar and elsewhere, a very high treatment coverage and compliance at national and local level is key and additional control measures such as snail control and behaviour change interventions will need to be implemented area wide.

**Trial Registration:**

ISRCTN48837681.

## Background

For almost a century, urogenital schistosomiasis has been recognised as a public health problem on the Zanzibar islands that form part of the United Republic of Tanzania [1–3]. The first programme to control morbidity of the disease dates back to 1986 on Pemba island [4]. Since then, considerable progress in the control of urogenital schistosomiasis and other neglected tropical diseases (NTDs), such as soil-transmitted helminthiasis and lymphatic filariasis, has been made [5–8]. Between 1994 and 1999, children and sometimes entire communities were treated annually with praziquantel for schistosomiasis and with albendazole for soil-transmitted helminthiasis [9]. In 2001, mass drug administration (MDA) against lymphatic filariasis was started and ivermectin and albendazole were distributed to all at-risk communities in Unguja and Pemba in six treatment rounds, the latest being implemented in 2006 [5, 9, 10]. Additionally, between 2003 and 2007, several hundred thousand schoolchildren were treated annually with praziquantel for schistosomiasis and with albendazole for soil-transmitted helminthiasis in the framework of the *Piga vita Kichocho* Programme and supported by the Schistosomiasis Control Initiative (SCI) on Unguja and Pemba islands [6]. After a period of intermittent treatment efforts between 2008 and 2011, biannual community-wide treatment (CWT) with praziquantel and albendazole were instituted in 2012 with the start of the Zanzibar Elimination of Schistosomiasis Transmission (ZEST) project, which aims to eliminate urogenital schistosomiasis transmission from Unguja island and to eliminate urogenital schistosomiasis as a public health problem from Pemba island [11, 12].

In the frame of ZEST, a multi-year randomised operational research project, funded by the Schistosomiasis Consortium for Operational Research and Evaluation (SCORE), was implemented to assess whether snail control and behaviour change interventions as complementary measures to MDA have a larger impact on *S. haematobium* prevalences and infection intensities than MDA alone [11, 12]. Since April 2012, CWT with praziquantel and albendazole is implemented twice a year by the NTD Control Programme of the Zanzibar Ministry of Health. School-based treatment (SBT) was introduced as an additional venue for MDA in November 2013, with an aim to further improve the coverage of school-aged children.

Achieving and maintaining high MDA coverage is essential for the success of helminth control and elimination programmes [13–15]. In countries aiming to control morbidity due to schistosomiasis and soil-transmitted helminthiasis, the World Health Organization (WHO) has set the target to reach 100 % geographical and at least 75 % national coverage [15, 16]. To achieve interruption of transmission and elimination of schistosomiasis, it is recommended to increase the frequency of treatment and to consolidate MDA by additional control measures such as snail control, behaviour change interventions and improved access to water and sanitation [15, 17, 18]. Coverage is also considered a minimum process indicator for assessing the performance of large-scale preventive chemotherapy interventions [13, 15]. To monitor the progress of helminthiasis control programmes and the success of MDA, programme managers are supposed to compile data recorded in registers by community drug distributors (CDDs) during MDA and to calculate the programme coverage based on the number of people treated divided by the number of persons at risk for the target disease [19]. National authorities are expected to submit their programme report, including the coverage achieved in their helminth control programmes to WHO on an annual basis [20]. In theory, programme coverage refers to the proportion of individuals in the target population who have actually swallowed the recommended drug or drug combinations in the designated endemic area [20]. In practice, coverage data are often calculated based on the number of tablets distributed to the target population [19]. Since the intake of the drugs cannot always be directly observed by CDDs, it is recommended to conduct random cluster surveys to assess the actual coverage including drug intake, here termed compliance [13, 21]. To validate the accuracy of country reported MDA coverage rates, it is recommended to conduct periodically post-MDA surveys implemented by trained interviewer teams that were not involved in the MDA implementation [14, 19].

We aimed to assess the SBT and CWT coverage with a post-MDA survey conducted within the annual parasitological survey of the SCORE study, 3–5 months after the SBT and CWT had been implemented on Pemba and Unguja islands in November 2013. Moreover, we wished to elucidate reasons for not receiving or taking praziquantel and, finally, we aimed to validate the accuracy of the coverage rates reported by the MoH according to data collected by local CDDs and to draw recommendations for improving future treatment coverage and schistosomiasis control and elimination efforts in Zanzibar and elsewhere. To substantiate our quantitative findings, we occasionally added quotes from a small qualitative inquiry that had been conducted with community members in August 2013 to identify potential barriers to taking praziquantel (unpublished report; Person, B. “A human centered design project. The Zanzibar elimination of schistosomiasis transmission study: year 2 qualitative research findings on the behavioral intervention”; December 2013).

## Methods

### Ethics statement

Ethical approval for the protocol of the randomised intervention trial “Study and implementation of schistosomiasis elimination in Zanzibar (Unguja and Pemba Islands) using an integrated multidisciplinary approach” funded by SCORE was obtained from the Zanzibar Medical Research Ethics Committee (ZAMREC; reference no. ZAMREC 0003/Sept/011), the “Ethikkommission beider Basel” (EKBB) in Switzerland (reference no. 236/11) and the Institutional Review Board of the University of Georgia (project no. 2012-10138-0). The study is registered at the International Standard Randomised Controlled Trial Number Register (ISRCTN48837681).

Before the onset of the annual parasitological survey including questionnaire interviews in March 2014, the district health and school authorities were informed about the aims and objectives of the study and about the survey dates. On the day of the survey, the headmaster of the school and the community leader (sheha) were visited by a senior member of the field team, who explained the purpose and procedures of the study verbally and asked for permission to carry out the survey in the school or community. The study was also explained in lay terms to the schoolchildren and adult participants, who additionally received an information sheet and a consent form to sign. Minors (e.g. children below the age of 16 years) were asked for written informed consent by their parent/guardian. All participants were offered praziquantel (40 mg/kg) for schistosomiasis and albendazole (400 mg) for soil-transmitted helminthiasis free of charge as part of the island-wide MDA round conducted in August 2014.

### Study area and population

Tanganyika and the Zanzibar islands established the United Republic of Tanzania in April 1964. The two main islands of the Zanzibar archipelago, Unguja and Pemba, are located approximately 30 km and 50 km, respectively, east of mainland Tanzania in the Indian Ocean. Both islands are divided into districts, which are further split into a total of 331 small administrative areas (shehias) [22]. According to the 2012 population and housing census, Pemba has 121 and Unguja 210 registered shehias, an approximate combined population of 1.3 million inhabitants and an average household size of 5.1 people [22, 23]. In 2012, there were 194 primary schools in Zanzibar. The net enrolment rate in primary schools was 83.7 %, with a net proportion of that population of 79.1 % and 4.6 % in public and private primary schools, respectively [24]. Urogenital schistosomiasis caused by *S. haematobium* was highly prevalent in Unguja and Pemba in the 1980s [4, 25, 26]. It is the only autochthonous form of schistosomiasis on the islands, since the only intermediate host snail species present is *Bulinus globosus* [27]. Social improvements and repeated MDA with praziquantel over the past decades have reduced prevalence and morbidity to low levels, and hence elimination of urogenital schistosomiasis seems feasible [8, 11, 28].

### Implementation of SBT and CWT in Zanzibar in November 2013

Biannual CWT has been implemented by the Zanzibar MoH as part of the strategy to eliminate urogenital schistosomiasis since April 2012 and followed the “3-year comprehensive strategic plan to combat neglected tropical diseases in Zanzibar 2009/2011”, referred to as the National Plan [11, 29]. The CWT conducted in November 2013 was the fourth treatment round. With the aim to achieve a higher coverage and compliance of the school-aged population, SBT was added as an additional venue in MDA and implemented for the first time in the framework of the ZEST project in November 2013. The SBT followed a directly observed treatment (DOT) approach and was conducted over several weeks by members of the NTD Control Programme of the Zanzibar MoH in close collaboration with teachers and shehas. All public primary schools in Pemba and all public primary schools in the North A, North B, Central, West and Urban C districts in Unguja were supposed to be targeted with albendazole and praziquantel. All schools were advised to provide porridge to the children on the day of SBT to reduce the frequency of potential drug-related adverse events [30–32]. The CWT was implemented across Pemba and Unguja islands on 29th and 30th November, 2013, by trained CDDs, supervised by members of the NTD Control Programme of the Zanzibar MoH and District Health Management Teams. The CDDs used a door-to-door approach to distribute albendazole and praziquantel to all people in Zanzibar, with the exception of children below the age of 3 years, children who had received treatment in the SBT, pregnant women and severely sick persons. Praziquantel was supposed to be distributed using a dose pole [33, 34]. Noteworthy, the exclusion of pregnant women from praziquantel treatment was based on local guidelines. The WHO recommends the treatment of pregnant and lactating women [13, 35] and efforts are currently underway in Zanzibar to adapt the local guidelines accordingly.

#### Study design

The post-MDA survey described here was embedded in the annual parasitological survey of the SCORE randomised operational research project, carried out from March to May 2014, 3–5 months after the SBT and CWT were conducted in Zanzibar in November 2013. In line with the SCORE study protocol, where the study design and sample size calculations are described in detail [11], a total of 45 schools and 45 shehias were included in the cross-sectional survey on Pemba island and a total of 48 schools and 47 shehias were surveyed on Unguja island. In each school, approximately 130 children from standards 3 and 4 were randomly selected to participate. In each shehia, 50 adults were randomly selected to participate. For analysis, the shehias and schools on each island served as primary sampling units.

### Field procedures

Before the onset of the study, all shehas and headmasters of the schools were informed about the purpose of the project and asked for their support during the study. According to the protocol [11], all children aged 9–12 years visiting the study schools were eligible to participate. This age group is primarily found in standards 3 and 4, and hence, in each primary school visited, the study was explained to children from these grades, before 130 children were randomly selected and invited to participate. For randomization, all eligible children lined up, in separate lines for boys and girls and grade. Subsequently, each third child in the lines was systematically selected to be included in the study. This procedure was continued until we reached a total of 130 selected children, accounting for a 20 % drop-out with the final aim to sample 100 children aged 9–12 years. The selected children were registered and their age, sex and participation at the last SBT were recorded. Subsequently, they received an information sheet and a consent form, which they were asked to return the following day with a signature of their parent/guardian. On the next day, children that provided a signed consent form were given a labelled transparent container (100 ml) with lid and invited to submit an own urine sample between 10:00 and 12:00 h.

In the shehias, we selected a quota sample of 50 houses per shehia according to a method suggested by Winkler et al*.* [36]. For randomisation, a gyro with a marked arrow-star (equal to the number of field interviewers in a team) pointing into different directions was spun on a central point in the shehia [11]. Subsequently, each interviewer counted the households to the edge of the shehia following a path in the direction of the arrow. On return to the centre point, the number of households in that direction was reported and the interviewer stated a number within the range of the counted households. The number was compared to a list with computer-generated random numbers created for each shehia. The random number corresponding to the number stated by the interviewer assigned where to start the first questionnaire interview. Subsequently, the neighbouring households were visited. In total, 50 individuals from 50 households were invited to participate. A household was defined as “a group of persons who normally live and eat together”. In case there was more than one household member present at the time when the fieldworker knocked at the door, a member was randomly selected by drawing cards. However, since generally more women than men were present at home at the time of the household visits, each fieldworker was advised that at least a third of the participants should be male. The age range for participation was 20–55 years, according to the SCORE study protocol [11]. After signing a consent form to participate, each individual was interviewed with a closed-ended questionnaire about the participation at the last CWT in November 2013, i.e. whether he/she knew, which disease was treated with praziquantel (a picture of different drugs distributed during the CWT, including praziquantel, was shown), whether he/she had received praziquantel, had been measured for height, had taken praziquantel, and had taken all praziquantel tablets together or split the intake. When people had not received or taken the tablets, the reason was recorded. Finally, the interviewee was invited to submit a urine sample for examination for a *S. haematobium* infection between 10:00 and 14:00 h.

### Laboratory procedures

All urine samples were brought back to the Helminth Control Laboratory on Unguja and the Public Health Laboratory—Ivo de Carneri on Pemba on the day of collection. Urine samples were inspected for microhaematuria using reagent strips (Hemastix; Siemens Healthcare Diagnostics GmbH, Eschborn, Germany) and for *S. haematobium* eggs by filtering 10 ml of urine through a polycarbonate filter (Sterlitech, Kent, United States of America) and subsequent quality controlled microscopy of the filter by experienced laboratory technicians.

### Data management and analysis

Registration details of pupils and laboratory results of children and adults participating in the SCORE annual parasitologal survey were entered into a Microsoft Excel spreadsheet (version 10.0; 2002 Microsoft Corporation). Questionnaire data of adults were entered into EpiInfo version 3.5.1 (Centers for Disease Control and Prevention, Atlanta, United States of America). Statistical analyses were carried out with STATA version 12 (StataCorp., College Station, United States of America). A Microsoft Excel database, including the numbers of children treated and registered in the schools as recorded by the NTD Control Programme team members and teachers who conducted the SBT and the number of people treated and recorded by the CDDs conducting CWT, stratified by school, shehia, district and island, was provided by the MoH.

Only children aged 9–12 years and adults aged 20–55 years, who provided a written informed consent, responded to the questions about their participation at the SBT or CWT and had a complete urine analysis (i.e. reagent strip assessment plus urine filtration) were included into the final analysis of our SCORE post-MDA survey.

An individual was considered microhaematuria-positive, when the reagent strip colour was coded trace, +, ++ or +++, according to the manufacturer’s instructions. An individual was considered as schistosomiasis-positive, when at least one *S. haematobium* egg was identified on the urine filter.

Coverage was calculated as follows: first, in our SCORE post-MDA survey in the schools and communities, we estimated the proportion of pupils and adults, respectively, who received praziquantel among those who were interviewed and included into our analysis. Second, we calculated the proportion of adults who had received and taken praziquantel among those who were interviewed and included into our analysis. Third, we excluded adults that might have been considered as not eligible for treatment by the MoH (pregnant, breastfeeding or ill) from the analysis and calculated the proportion of individuals who had received praziquantel. Fourth, we again excluded potentially not eligible (pregnant, breastfeeding or ill) adults from the analysis and calculated the proportion of individuals who had received and taken praziquantel. Fifth, with regard to the data obtained from the MoH, we calculated coverage as the proportion of treated individuals among the total population as recorded by the CDDs. Sixth, we calculated coverage as the proportion of treated individuals among the population considered as eligible by the MoH. Country-reported coverage data were considered accurate if they fell within the 95 % confidence intervals (CI) of the survey coverage rates [19]. Prevalences and coverage rates, including 95 % CIs, were calculated taking into account the cluster survey design and using *svyset* and *svy:* commands in Stata. Design effects were estimated for the survey coverage rates. Risk factors for *S. haematobium* infection as outcome (binary) were assessed with multivariable regression analysis, using treatment (binary), sex (binary) and age (continuous in years) as explanatory.

## Results

### Study participation

**Fig. 1:**
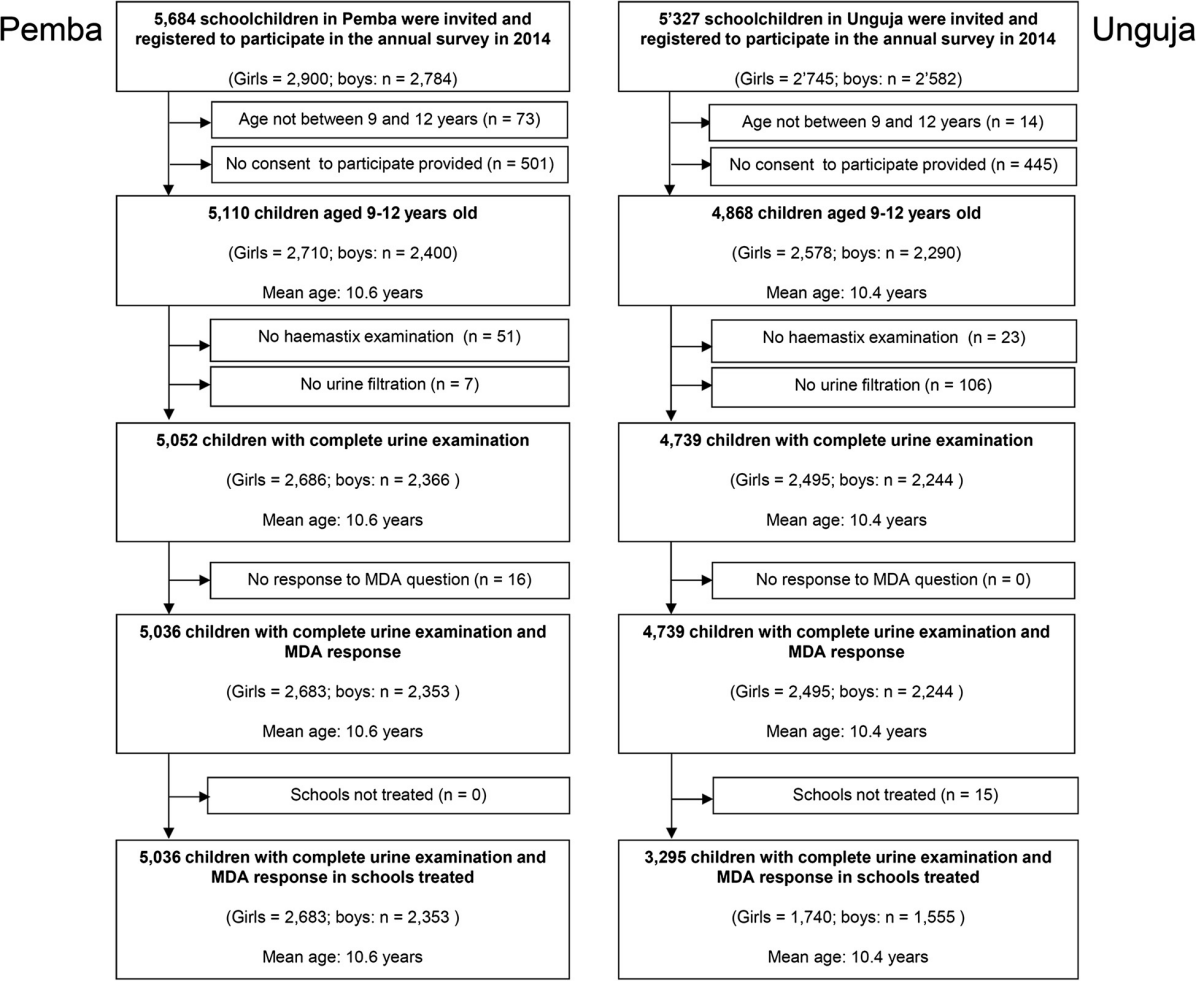
Participation of 9- to 12-year-old pupils in the post-mass drug administration survey conducted in Zanzibar

As shown in Fig. 1, a total of 5684 and 5327 pupils in Pemba and Unguja, respectively, were invited and registered to participate in our parasitological survey. In Pemba, a total of 5036 children from all 45 SCORE schools had complete data and were included in the analysis. Among them, 2683 were girls and 2353 were boys. In Unguja, complete data were available from 4739 pupils. However, among them, 1444 children attended one of 15 schools that were not targeted by the SBT and were excluded from further analysis. Ultimately, in Unguja, a total of 3295 children were included in the final analysis. Among them, 1740 were girls and 1555 were boys.

**Fig. 2:**
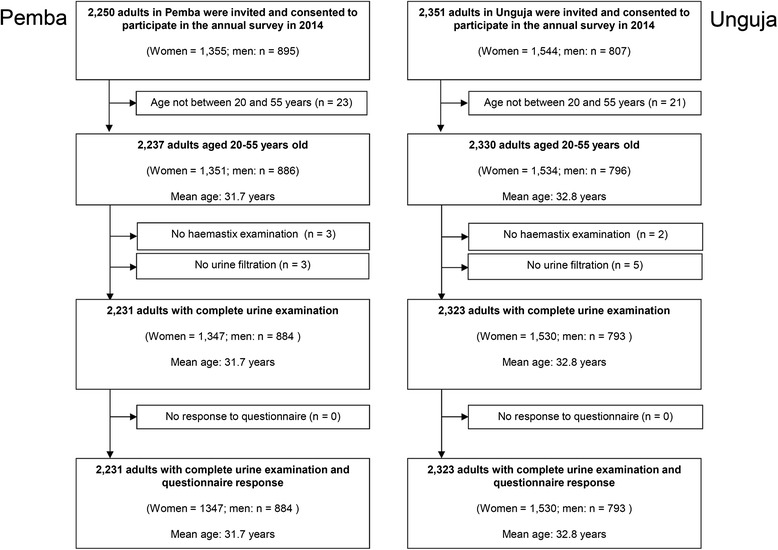
Participation of 20- to 55-year-old adults in the post-mass drug administration survey conducted in Zanzibar

Figure 2 shows that a total of 2250 and 2351 adults in Pemba and Unguja, respectively, consented to participate in our study. Inclusion criteria were fulfilled by 2231 adults in Pemba and by 2323 adults in Unguja. Among them, 60.4 % in Pemba and 65.9 % in Unguja were female.

### Praziquantel treatment coverage in SBT and CWT according to the SCORE post-MDA survey

In Pemba, SBT had been carried out in all 45 SCORE schools. As shown in Table 1, 85.8 % of interviewed pupils reported that they had taken praziquantel during the SBT. The remaining children stated that they were not present at the SBT and had not received praziquantel during the CWT. The coverage was higher in girls (88.8 %) than in boys (82.4 %). In Unguja, only children from 33 among the 48 schools included in the annual SCORE survey reported that they had participated at the SBT. Among the children that attended one of the 33 covered schools, 83.1 % reported that they had ingested praziquantel during the SBT. The coverage was similar among girls (83.9 %) and boys (82.1 %).

**Table 1:** Coverage and compliance in school-based and community-wide treatment carried out in Zanzibar in November 2013

	Unguja		Pemba	
	n	%	n	%
School survey				
Schools	33 (of 48)		45 (of 45)	
Pupils	3295		5036	
Boys	1555		2353	
Girls	1740		2683	
Did not receive or swallow praziquantel	558	16.9	715	14.2
Boys	278		415	
Girls	280		300	
Received and swallowed praziquantel	2737	83.1	4321	85.8
Boys	1277	82.1	1938	82.4
Girls	1460	83.9	2383	88.8
Adult survey				
Shehias	47 (of 47)		45 (of 45)	
Adults	2323		2231	
Men	793		884	
Women	1530		1347	
Did not receive praziquantel (or did not know if received)	804	34.6	1035	46.4
Men	263	33.2	422	47.7
Women	541	35.4	613	45.5
Received praziquantel	1519	65.4	1196	53.6
Men	530	66.8	462	52.3
Women	989	64.6	734	54.5
If received praziquantel: Took all praziquantel together	1263	83.1	977	81.7
Men	433	81.7	391	84.6
Women	830	83.9	586	79.8
If received praziquantel: Did not take praziquantel or did not know	256	16.9	219	18.3
Men	97	18.3	71	15.4
Women	159	16.1	148	20.2
Did not receive or did not take praziquantel	1060	45.6	1254	56.2
Men	360	45.4	493	55.8
Women	700	45.8	761	56.5
Received and took all praziquantel together	1263	54.4	977	43.8
Men	433	54.6	391	44.2
Women	830	54.2	586	43.5
Received and took all praziquantel together and were measured for height	913	39.3	602	27.0
Men	298	37.6	221	25.0
Women	615	40.2	381	28.3

In Pemba, among the 2231 interviewed adult participants, 53.6 % confirmed that they had received praziquantel in the CWT. However, only 43.8 % had swallowed all received tablets together. Considering that only 602 adults who had received and taken all praziquantel tablets together had also been measured for height, a proper intake of drugs can be assumed for 27.0 % of adults.

In Unguja, among the 2323 adults included in the analysis, 65.4 % reported that they had received praziquantel from a CDD and 54.2 % had taken the drugs. However, height had only been measured for 913 individuals. Hence, a proper praziquantel intake can be assumed for 39.3 % of adults.

Noteworthy, among adults interviewed in Pemba and Unguja, only 69.3 % and 36.5 %, respectively, recognised praziquantel as the drug to treat schistosomiasis. In Pemba, 29.8 % and in Unguja 49.6 % of adults stated they did not know which disease was treated with the received tablets. Intestinal worms, lymphatic filariasis, malaria and eye disease were incorrectly mentioned as diseases that could be treated with praziquantel.

### Reasons for not receiving or taking praziquantel in the CWT

In Pemba, 1254 adults did not receive or did not take all praziquantel tablets together. Among them, 92.2 % provided at least one reason for not having received or taken the drug (Table 2). Reasons including the local treatment guideline exclusion criteria applied by the MoH were pregnancy (16.7 %), breastfeeding (5.8 %) and illness (2.6 %). Other main factors were absenteeism (39.3 %), being busy (7.6 %), no CDD coming to the house (6.6 %), being afraid of adverse events (6.4 %) or considering themselves as healthy (6.1 %).

**Table 2:** Reasons for not receiving or taking praziquantel during community-wide treatment in Zanzibar in 2013

	Unguja		Pemba	
	n	%	n	%
Either did not receive or did not take the drug	1060		1254	
Gave at least one reason for not receiving or taking the drug	699		1156	
Did not provide any reason	361	34.1	98	7.8
Reason provided:				
Pregnant	163	23.3	193	16.7
Breastfeeding	36	5.2	67	5.8
Sick	27	3.9	30	2.6
Absent < 5 days	119	17.0	228	19.7
Absent > 5 days	174	24.9	226	19.6
No CDD	104	14.9	76	6.6
No praziquantel left	13	1.9	11	1.0
Busy	12	1.7	88	7.6
Afraid of adverse events	16	2.3	74	6.4
Healthy	13	1.9	70	6.1
Too many tablets	2	0.3	41	3.5
Did not trust height measurement	3	0.4	25	2.2
No information about the drugs	8	1.1	16	1.4
Did not like the drug	2	0.3	14	1.2
No food available	1	0.1	9	0.8
Kept	3	0.4	7	0.6
Drug does not work	1	0.1	6	0.5
Bad CDD	0	0.0	5	0.4
Drug does affect reproduction	0	0.0	3	0.3
Fasting	3	0.4	1	0.1
Too old	2	0.3	0	0.0
Too young	0	0.0	0	0.0
Religion	0	0.0	0	0.0
Drug provided for free	0	0.0	0	0.0

*CDD* Community Drug Distributor

In Unguja, only 65.9 % among the 1066 adults who had not received or taken all praziquantel tablets together provided at least one reason for the non-compliance: pregnancy, breastfeeding and being ill were mentioned by 23.3 %, 5.2 % and 3.9 %; absenteeism on the days of CWT was reported from 41.9 % and no CDD visiting the house was stated by 14.9 % of adults.

In our earlier qualitative inquiry, people described difficulties in taking the large number of tablets provided during the CWT. A 51-year-old health worker from Pemba said, *“I suggest that you should divide the tablets into two parts because some people are weak they cannot afford to take a lot of drugs at the same time. It would also avoid complications or drug reactions”. (Int. 11)*. A 44 year-old teacher from Unguja told us, *“You know, for our people it is difficult to swallow many tablets at the same time because of their health. If we are given nearly ten tablets—tablets for the helminths, filariasis and these kichocho [schistosomiasis] tablets, it would not be good. I think the government should reduce the number of the tablets as we know our people. They will take the tablets but not swallow them”. (Int. 50).*

Adults and children also expressed concerns over drug-related adverse events. A woman’s group leader reported, *“I knew they [children] had received the drugs because they came home and told me. They came home complaining about abdominal discomfort after being given the drugs at school. They are given the drugs after every six or so months…am not sure….but I know they are given the drugs at school”. (Int. 65)* Another person reported, *“These tablets are powerful so some people get dizziness when they swallow them. So they are afraid. Some of them they get vomiting too”. (Int. 97)*

### Coverage reported by MoH versus results from the SCORE post-MDA survey

**Fig. 3:**
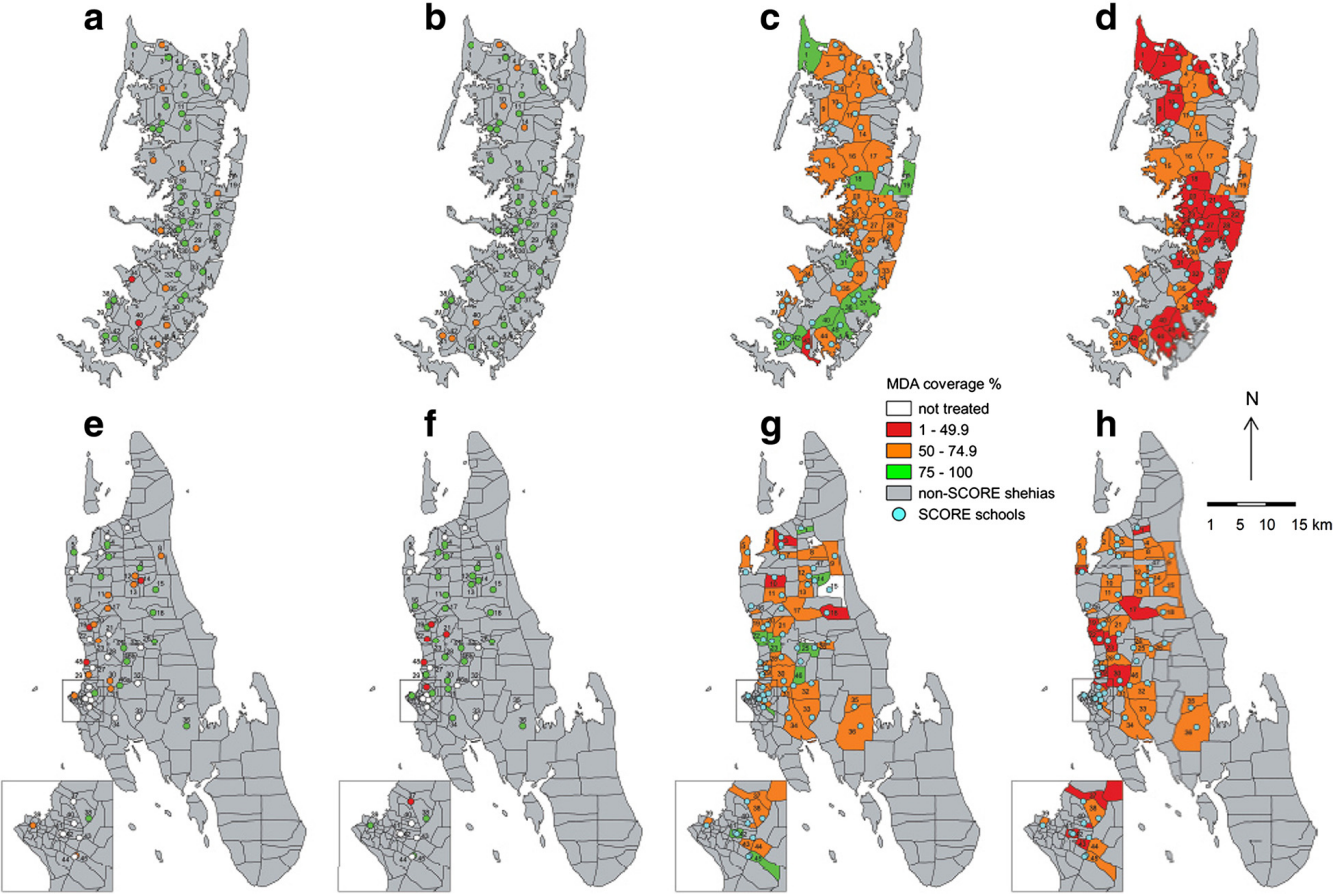
Coverage rate achieved with school-based (SBT) and community-wide treatment (CWT) in Zanzibar in November 2013. Key to colours: White: not treated or no/invalid data; Red: coverage of 1–49 %; Orange: coverage of 50–74 %; Green: coverage of 75–100 %. **a** Pemba coverage of SBT according to the Zanzibar Ministry of Health (MoH); no data were available for the schools of two shehias in Pemba. **b** Pemba coverage of SBT according to our post-mass drug administration survey. **c** Pemba coverage of CWT according to the Zanzibar MoH. **d** Pemba coverage of CWT according to our post-mass drug administration survey. **e** Unguja coverage of SBT according to the Zanzibar MoH; no data were available for the schools of 19 shehias in Unguja. **f** Unguja coverage of SBT according to our post-mass drug administration survey (schools of 15 shehias indicated no treatment in children attending standards 3 and 4). **g** Unguja coverage of CWT according to the Zanzibar MoH (no data were available for 2 shehias and invalid data were available for another 2 shehias in Unguja). **h** Unguja coverage of CWT according to our post-mass drug administration survey. Shehias in Pemba (maps a–d): 1. Makangale; 2. Msuka; 3. Konde; 4. Kinowe; 5. Tumbe; 6. Mgogoni; 7. Shumba Viamboni; 8. Sizini; 9. Kizimbani; 10. Kinyasini; 11. Finya; 12. Selemu; 13. Jadida; 14. Pandani; 15. Mtambile Kusini; 16. Piki; 17. Mchangamdogo (no MoH school data); 18. Kisiwani; 19. Kangagani; 20. Ziwani; 21. Ole; 22. Uwandani; 23. Mbuzini; 24. Kwale; 25. Wesha; 26. Tibrinzi; 27. Ng’ambwa; 28. Vitongoji; 29. Wawi; 30. Chanjaani; 31. Shungi (no MoH school data); 32. Matale; 33. Pujini; 34. Wambaa; 35. Ngwachani; 36. Ukutini; 37. Chambani; 38. Makombeni; 39. Ng’ombeni; 40. Mtambile; 41. Michenzani; 42. Mkanyageni; 43. Kangani; 44. Kengeja; 45. Kiwani. Shehias in Unguja (maps e–h): 1. Mkwajuni; 2. Donge Mchangani; 3. Pale; 4. Gamba; 5. Mafufuni; 6. Makoba; 7. Donge Mtambile; 8. Chaani Masingi; 9. Kandwi; 10. Donge Mnyimbi; 11. Mahonda; 12. Bandamaji; 13. Mgambo; 14. Kinyasini; 15. Upenja; 16. Fujoni; 17. Kitope; 18. Kilombero; 19. Kama; 20. Mfenesini; 21. Mwakaje, 22. Chuini; 23. Mbuzini; 24. Miwani; 25. Kiboje Mkwajuni; 26. Uzini; 27. Mwanyanya (for e and f shehia highlighted since school coordinates not available); 28. Dole; 29. Mtoni; 30. Kianga; 31. Mwera shehia / Regeza Mwendo school; 32. Ubago; 33. Jumbi; 34. Fuoni Kibondeni; 35. Jendele; 36. Cheju; Inset map: 37. Mtopepo; 38. Welezo; 39. Mchangani; 40. Kilimahewa Juu; 41. Muungano; 42. Sebleni; 43. Nyerere; 44. Mwanakwerekwe; 45. Melinne; 46. Koani; 46.a Machui school (Koani shehia); 46.b Mwera school (Koani shehia); 47. Chaani Mcheza Shauri (only CWT maps g and h); 48. Bububu school (only SBT maps e and f)

The maps in Fig. 3 show the observed and reported coverage rates achieved with SBT and CWT in Pemba and Unguja, stratified into high (≥75.0 %), moderate (50.0–74.9 %) and low (<50.0 %) coverage. In regard to SBT, in Pemba, 43 among the 45 SCORE schools were included in the MoH database and could be compared to our SCORE post-MDA survey. In these schools, the MoH reported a SBT coverage of 84.3 % and our survey revealed a coverage of 85.2 % (95 % CI: 81.8–88.6 %) (Table 3).

**Table 3:** Reported and observed coverage of school-based and community-wide treatment in Zanzibar in 2013. Treatment coverage according to community drug distributor reports to the Ministry of Health (MoH) and coverage and compliance according to the Schistosomiasis Consortium for Operational Research and Evaluation (SCORE) post-mass drug administration (MDA) survey carried out as part of a parasitological survey from March to May 2014, 3–5 months after the school-based and community-wide treatment with praziquantel in November 2013 on Unguja and Pemba islands, United Republic of Tanzania

		MoH data			SCORE post-MDA survey								
	Number treated/number surveyed	Population	Treated population	% Treated	Surveyed population	Received treatment	Received and swallowed tablets	% Received treatment	95 % CI	Design effect	% Received and swallowed tablets	95 % CI	Design effect
Unguja													
Schools: registered population	27^c^/48	26,612	17,005	63.9	2754		2394				86.9	81.0–92.9	20.10
Shehias: total population	43^b^/47	181,434	122,860	67.7	2123	1378	1147	64.9	60.7–69.1	4.0	54.0	50.3–57.7	2.9
Shehias: eligible population^a^	43^b^/47	152,362	122,860	80.6	1907	1358	1147	71.2	66.6–75.8	4.9	60.1	55.9–64.4	3.5
Pemba													
Schools: registered population	43^d^/45	28,235	23,807	84.3	4803		4092				85.2	81.8–88.6	10.7
Shehias: total population	45/45	224,518	132,475	59.0	2231	1196	977	53.6	50.4–56.8	2.2	43.8	40.5–47.1	2.4
Shehias: eligible population^a^	45/45	161,597	132,475	82.0	1940	1166	976	60.1	56.5–63.7	2.6	50.3	46.6–54.0	2.7

95 % CI: 95 % confidence intervals

^a^People who were sick, pregnant or breastfeeding were excluded from analysis

^b^Four shehias were excluded from analysis due to problems with data obtained from the MoH (Upenja had duplicate, Miwani had invalid, and Gamba and Makoba had no MoH data)

^c^21 schools were excluded from analysis. Reason: no MoH data were available for 19 schools in Unguja (among them, 13 schools also did not report any treatment in the post-MDA survey) and 2 additional schools, which reported no treatment in the post-MDA survey

^d^Two schools were excluded from analysis. Reason: no MoH data were available for the schools of two shehias in Pemba (Shungi and Mchanga Mdogo)

In Unguja, only 27 among the 48 SCORE schools had treatment reports from both, MoH and our SCORE post-MDA survey. Among the 21 “missing” schools, 13 had reported that they were not targeted by the SBT in our post-MDA survey and were also not listed in the MoH database; six schools had indicated SBT in our post-MDA survey but were not listed in the MoH database; and two schools were listed in the MoH database but no child from standards 3 and 4 had indicated treatment in our survey. In the 27 “available” schools, the coverage was 63.9 % according to MoH data and 86.9 % (95 % CI: 81.0–92.9 %) according to the SCORE post-MDA survey.

In regard to the CWT, in Pemba, all 45 SCORE shehias were listed in the MoH database. Considering the total population recorded by the CDDs as denominator, the MoH data resulted in a coverage of 59.0 %, while the SCORE post-MDA survey showed that 53.6 % (95 % CI: 50.4–56.8 %) of the participants had received praziquantel, but that only 43.8 % (95 % CI: 40.5–47.1 %) had taken all drugs together. If the population recorded by the CDD as eligible was taken as denominator, the MoH reported coverage was 82.0 %. If pregnant, lactating and ill people were excluded from the SCORE post-MDA survey analysis, 60.1 % (95 % CI: 56.5–63.7 %) of adults had received praziquantel, but only 50.3 % (95 % CI: 46.6–54.0 %) had taken all tablets together.

In Unguja, among the 47 shehias surveyed for the SCORE post-MDA survey, 43 had valid entries in the MoH database. The praziquantel coverage in the total population was 67.7 % according to the CDD reports and 64.9 % (95 % CI: 60.7–69.1 %) in our survey. However, only 54.0 % (95 % CI: 50.3–57.7 %) of our study population had taken all praziquantel tablets together. Considering only the eligible population, the reported and observed coverage was 80.6 % and 71.2 % (95 % CI: 66.6–75.8 %), respectively and 60.1 % (95 % CI: 55.9–64.4 %) had finally taken all tablets together.

### Prevalence of *S. haematobium* in the study population

In Pemba, the overall prevalence of microhaematuria and *S. haematobium* infection in the 45 schools was 7.4 % (95 % CI: 5.4–9.5 %) and 5.4 % (95 % CI: 3.4–7.3 %), respectively. Treated children had significantly lower odds than untreated children (OR: 0.5; 95 % CI: 0.3–0.9 %) and boys had significantly higher odds than girls (OR: 1.8; 95 % CI: 1.3–2.6 %) of being infected with *S. haematobium*.

In Unguja, the overall prevalence of microhaematuria and *S. haematobium* infection in the 33 treated schools was 7.4 % (95 % CI: 4.6–10.3 %) and 5.2 % (95 % CI: 1.9–8.5 %), respectively. In the 15 schools that were not covered by SBT, the overall prevalence of microhaematuria was 3.7 % (95 % CI: 2.5–4.9 %) and of *S. haematobium* infection was 2.2 % (95 % CI: 0.8–3.6 %). Boys had significantly higher odds of harbouring a *S. haematobium* infection than girls (OR: 1.7; 95 % CI: 1.1–2.7 %).

In the adults residing in one of the 45 SCORE shehias in Pemba, the overall prevalence of microhaematuria and *S. haematobium* infection was 19.4 % (95 % CI: 17.2–21.5 %) and 4.0 % (95 % CI: 2.5–5.4 %), respectively. An increase of age significantly lowered the risk of being infected with *S. haematobium* (OR: 0.95, 95 % CI: 0.93–0.97 %).

In Unguja, 10.8 % (95 % CI: 9.3–12.4 %) of the adult population was microhaematuria-positive and 2.9 % (95 % CI: 1.7–4.0 %) excreted *S. haematobium* eggs. Men had higher odds (OR: 1.9; 95 % CI: 1.1–3.5 %) and older people lower odds (OR: 0.92; 95 % CI: 0.88–0.96 %) of being infected with *S. haematobium*.

## Discussion

Achieving and maintaining high MDA coverage is key for the success of helminthiases control and elimination programmes. We found that the SBT coverage on Pemba and Unguja islands was 85.2 % and 86.9 %, respectively, and hence in line with MoH reports from Pemba (84.3 %) and higher than reports from Unguja (63.9 %). However, in Unguja, around one third of the visited schools had not been covered by SBT at all and was excluded from this analysis. The CWT compliance observed in our post-MDA survey was significantly lower than the coverage reported by the MoH. While according to the CDD reports more than 80 % of the eligible population had received treatment in the CWT, we found that only 71.2 % and 60.1 % of the eligible adult population in Unguja and Pemba, respectively, had received praziquantel and that only 60.1 % and 50.3 %, respectively, of the eligible adult population complied with the praziquantel intake. In our study population, the main reasons for not having received or taken the drugs were absence during CWT, no drug distributor coming, being busy, fear of adverse events, pregnancy, breastfeeding or feeling healthy.

Our findings have important implications for the schistosomiasis elimination project in Zanzibar. While more than 75 % of pupils were treated during the SBT coverage in the targeted schools, the MoH will need to make an effort to reach out to all schools in Zanzibar, including all public and private schools, nurseries and madrassas (Koran schools). One reason for not reaching all schools during the November 2013 treatment in Unguja was that the SBT was conducted during the examination period and thus access to schools was restricted, children could not be treated during examination or were absent when not participating in examinations. Timing of the SBT is hence crucial for achieving high national coverage. The high coverage (>75 %) achieved in most of the targeted schools is likely due to the close supervision of teachers by the members of the NTD Control Programme team and the provision of porridge to children before treatment, which might have motivated them to come to school and mitigated the fear and occurrence of adverse events as suggested elsewhere [31, 32, 37].

The low coverage and compliance achieved during the CWT in Zanzibar as revealed in our post-MDA survey including adults shows that the approach to send CDDs to the houses was not very successful and that the CDD’s records over-reported treatment. A limitation of our study is that we did not assess the CDD’s motivation to distribute the drugs, the training they received or oversight and supervision for reporting. Further qualitative exploration is needed to better understand these gaps.

A lack of respect for the CDD, the smell and size of the tablets and the use of height, instead of weight, to determine the dosage, and the fear of side effects, infertility or even death have discouraged people from taking the drugs in previous studies conducted elsewhere in Africa [38–40]. In our survey, these issues were raised by few people, who had not taken the drugs. However, many people did not state a reason at all, and if a reason was given, it was mostly that people were absent from home on the days of CWT, were pregnant or breastfeeding. One might correctly argue that mobilisation and sensitisation of the community needs to be improved in future CWT in Zanzibar to achieve a better drug coverage. Moreover, pregnant and lactating women should be included into the treatment [13]. The implementation of robust qualitative inquiries could uncover more detailed reasons, which discourage community members from swallowing the tablets and also highlight how best to encourage community involvement in CWT and SBT. Our study showed that the knowledge of what disease was treated with praziquantel and about the necessity of taking tablets in the correct amount was only moderate to low in the adult population. Hence, better theory-based, culturally appropriate health communication about schistosomiasis and the negative health impact of schistosomiasis is critical.

However, we also need to challenge the approach of CWT for elimination purposes. After decades of fairly regular MDA in Zanzibar, prevalences and infection intensities of urogenital schistosomiasis have dropped to very low levels or zero in many areas, while high prevalences persist or rebound in other “hot-spot” areas [6, 7, 12]. The regular administration of drugs might have caused treatment fatigue in people and, particularly in areas of very low endemicity the inducement to receive and take the antischistosomal drug is low, when considering the daily income loss when waiting for a CDD. Moreover, the large-scale distribution of praziquantel to a population that is only marginally infected with *S. haematobium* is debatable, particularly since funds for drug distribution are limited. Elimination efforts might be more successful and cost-effective when CWT is focussed to hot-spot areas and a rigorous surveillance and response strategy is implemented in all health facilities across the islands [41]. For surveillance in Zanzibar, routine diagnosis, for example with reagent strips, will need to be implemented in public health care units, dispensaries, public health care centres and general hospitals and, for response, praziquantel treatment be made available in these peripheral centres and provided to suspected and confirmed cases. Since praziquantel treatment is also considered safe in pregnancy [42, 43], but often not received or taken by pregnant or lactating women, it will also be important to expand and improve the service of guidance, diagnosis and treatment to mother and child health care units.

On the other hand, previous research in Pemba has shown that the *S. haematobium* prevalence is underestimated when diagnosed by a single urine filtration and that considerably higher prevalences are revealed when an ultra-sensitive diagnostic test such as the up-converting phosphor lateral flow circulating anodic antigen (UCP-LF CAA) assay, is used [44]. Hence, unrecognised and untreated children and adults might constitute important parasite reservoirs that contribute to the spread of the disease and rapid reinfection [39, 45]. Considering that our post-MDA survey was conducted only 3–5 months after the last SBT and CWT and that a considerable number of treated individuals (4.7 % of children and 3.4 % of adults) were found to be *S. haematobium*-positive, the reinfection potential in certain hot-spot communities in Zanzibar seems to be relatively high, although incomplete cure by praziquantel might also be a plausible explanation [7, 46–48]. Achieving and maintaining high MDA coverage and compliance at national and local level and additionally treating infected individuals in health facilities are therefore crucial to avoid a rebound of *S. haematobium* infection. Highly sensitive diagnostic tests and additional control measures such as snail control and behaviour change interventions will be needed to interrupt transmission completely [18, 44, 49, 50]. The possibility of emerging drug resistance in Zanzibar, where praziquantel has been distributed regularly for more than 10 years, needs to be clarified and hopefully ruled out in future studies assessing praziquantel efficacy.

Limitations of our study are that, while our interviewers were trained and the questionnaires pretested, reporting bias cannot be ruled out completely and respondents might have provided socially desirable responses or might not have been able to recall treatment correctly. Moreover, our interviewers were not fully independent MoH staff but part of the NTD Control Programme team that implemented the MDA. Since, however, our results suggest an over-reporting of CWT coverage by the CDDs, we feel that the data collected in our post-MDA survey are reliable. For information on the SBT, we only included children from standards 3 and 4 in our survey, and results might not be generalisable for the whole schools. Finally, the schools and shehias investigated in our survey were not selected newly at random but comprised the settings included and annually surveyed in the SCORE randomised operational research project, which might have introduced a bias. A future post-MDA survey conducted by independent staff and in a sample of schools and communities that are selected newly and completely at random might shed light on the situation across the islands and show if the settings targeted by the SCORE operational research trial differ from the remaining schools and shehias in Zanzibar.

## Conclusion

To strengthen the elimination efforts in Zanzibar, SBT will need to be extended to all public and private schools, nurseries and madrassas. To overcome potential treatment fatigue in the communities and to increase coverage and compliance in adults, the implementation of the CWT needs to be improved and mobilization and sensitization enhanced. To reach elimination of urogenital schistosomiasis transmission in Zanzibar and elsewhere, achieving and maintaining very high treatment coverage and compliance at national and local level is key and additional control measures such as snail control and behaviour change interventions will need to be implemented area wide.
